# Draft Genome Sequence of *Leucobacter* sp. Strain UCD-THU (Phylum *Actinobacteria*)

**DOI:** 10.1128/genomeA.00325-13

**Published:** 2013-06-06

**Authors:** Hannah E. Holland-Moritz, Dakota R. Bevans, Jenna M. Lang, Aaron E. Darling, Jonathan A. Eisen, David A. Coil

**Affiliations:** University of California Davis Genome Center, Davis, California, USA

## Abstract

Here we present the draft genome of *Leucobacter* sp. strain UCD-THU. The genome contains 3,317,267 bp in 11 scaffolds. This strain was isolated from a residential toilet as part of an undergraduate project to sequence reference genomes of microbes from the built environment.

## GENOME ANNOUNCEMENT

Members of the *Leucobacter* genus are yellow-pigmented, Gram-positive, aerobic, nonmotile, non-spore-forming, irregular rod-shaped bacteria (1). They can be found in a variety of environments, including nematodes (2), waste treatment facilities (3), sediments receiving chromium-contaminated water (4), and cow dung (5).

*Leucobacter* sp. strain UCD-THU was isolated from a residential toilet in Davis, California, in an effort to provide reference genomes of microbes that are found in the built environment (6, 7). Biofilm scrapings were incubated overnight in Luria broth (LB) at 37°C, then plated out on LB agar. Single colonies were picked for serial dilution streaking and the organism was identified by Sanger sequencing of the 16S rRNA gene after PCR amplification using the 1391R and 27F primers. Genomic DNA was extracted using a Wizard genomic DNA purification kit (Promega) from fresh overnight cultures. Illumina paired-end libraries were then made from sonicated DNA using a TruSeq DNA sample prep v2 kit (Illumina). Fragments between 300 and 600 bp were selected using a Pippin Prep DNA size selection system (Sage Science).

A total of 3,121,654 paired-end reads were generated on an Illumina MiSeq, at a read length of 160 bp. Quality trimming and error correction of the reads resulted in 2,942,884 high-quality reads. All sequence processing and assembly were performed using the a5 assembly pipeline. This pipeline automates the processes of data cleaning, error correction, contig assembly, scaffolding, and quality control. The assembly produced 103 contigs, contained in 11 scaffolds (minimum, 5,587 bp; maximum, 699,082 bp; N_50_, 443,492 bp). During scaffolding, some contigs were merged based on short overlaps and read-pair information, yielding a final collection of 30 contigs in 11 scaffolds that were submitted to GenBank. This resulted in a final assembly of 3,317,267 bp with a GC content of 70% and an overall coverage estimate of 280×. Completeness of the genome was assessed using the Phylosift software (A. E. Darling, G. Jospin, E. Lowe, E. Matsen, H. Bik, and J. A. Eisen, submitted), which searches for a list of 40 highly conserved, single-copy marker genes (D. Wu, G. Jospin, and J. A. Eisen, in preparation), of which all 40 were found in this assembly.

Automated annotation was performed using the RAST annotation server (8). *Leucobacter* sp. strain UCD-THU contains 3,018 predicted coding sequences and 52 predicted RNAs. A full-length (1,488-bp) 16S sequence was obtained from this annotation and was used to attempt to identify the species of *Leucobacter*. The top 250 BLAST hits were aligned with MUSCLE (9), which was used to construct a phylogenetic tree with FastTree 2 (10) (doi:10.6084/m9.figshare.674598). *Leucobacter* sp. strain UCD-THU is most closely related to a single isolate, *L. chironomi*, but is more than 99% identical to several other *Leucobacter* species; therefore, were are unable to assign a species name to this isolate.

The genome sequence of only one other *Leucobacter* species has been published—that of *L. chromiiresistens* (11); this species also possesses a 16S rRNA gene sequence that is 99% identical to *Leucobacter* sp. strain UCD-THU.

### Nucleotide sequence accession numbers.

This whole-genome shotgun project has been deposited at DDBJ/EMBL/GenBank under the accession number APJM00000000. The version described in this paper is the first version, APJM01000000. Illumina reads are available at doi:10.6084/m9.figshare.201772.
